# Upregulation of Peroxiredoxin-2 in Well-Differentiated Pancreatic Neuroendocrine Tumors and Its Utility as a Biomarker for Predicting the Response to Everolimus

**DOI:** 10.3390/antiox9111104

**Published:** 2020-11-09

**Authors:** Eui Joo Kim, Yoon Jae Kim, Hye In Lee, Seok-Hoo Jeong, Hyo Jung Nam, Jae Hee Cho

**Affiliations:** 1 Division of Gastroenterology, Department of Internal Medicine, Gil Medical Center, College of Medicine Gachon University, Incheon 21565, Korea; imejkim21@gmail.com (E.J.K.); yoonmed@gachon.ac.kr (Y.J.K.); butterfly_77@naver.com (H.J.N.); 2 Division of Gastroenterology, Department of Internal Medicine, Gangnam Severance Hospital, Yonsei University College of Medicine, Seoul 06273, Korea; wnsgpdls@hanmail.net; 3 Division of Gastroenterology, Department of Internal Medicine, Catholic Kwandong University International St. Mary’s Hospital, Incheon 22711, Korea; ssukoo@naver.com

**Keywords:** pancreatic neuroendocrine neoplasm, peroxiredoxin, mTOR inhibitor, everolimus

## Abstract

Pancreatic neuroendocrine neoplasms (pNENs) account for 2–3% of pancreatic malignancies. Peroxiredoxins (Prdxs), which are major cellular antioxidants, are involved in multiple oncogenic signaling pathways. We investigated the role of peroxiredoxin-2 in QGP-1 human pNEN cell line and patient-derived pNEN tissue. To validate the cancer stem cell-like cell characteristics of QGP-1 cells in spheroid culture, in vitro analyses and xenografting were performed. Furthermore, immunohistochemical staining was conducted to verify the overexpression of Prdx2 in pNEN tissue. Prdx2 expression was high at the mRNA and protein levels in QGP-1 cells. Prdx2 was also overexpressed in patient-derived pNEN tissue. Silencing of Prdx2 using siRNA induced overexpression and phosphorylation of ERK and AKT in QGP-1. Cell proliferation was increased by treating QGP-1 cells with siPrdx2, and the IC50 of everolimus increased suggesting resistance to everolimus. Interestingly, QGP-1 spheroid cells, which exhibited cancer stem cell-like features, exhibited lower expression of Prdx2 and mTOR. The results suggest that Prdx2 expression level and its activity may be a potential predictive biomarker for therapeutic response or resistance to everolimus in pNEN.

## 1. Introduction

Pancreatic neuroendocrine neoplasms (pNENs), previously known as islet cell tumors or pancreatic endocrine tumors, are rare neoplasms accounting for approximately 2–3% of primary pancreatic malignancies [1,2]. Compared to pancreatic ductal adenocarcinomas, pNENs exhibit slower growth, and the prognosis is dependent on the histological classification. The World Health Organization, in 2017, classified pNENs into well-differentiated pNENs and poorly differentiated pancreatic neuroendocrine carcinomas (pNECs), and categorized pNENs as low grade (G1), intermediate grade (G2), or high grade (G3) based on mitotic activity or the Ki-67 labeling index [3]. Chemotherapy is the standard treatment for poorly differentiated pNECs, but well-differentiated pNENs require various therapeutic methods including surgical resection, somatostatin analogues, the mechanistic target of rapamycin (mTOR) inhibitor everolimus, the multikinase inhibitor sunitinib, and peptide receptor radiotherapy. Although surgery is the only curative method for these patients, because most are diagnosed at an advanced stage, the five-year survival rate is reported to be approximately 32% [3,4]. Therefore, the molecular features associated with poor prognosis need to be determined, and more effective treatments for pNEN are required.

Peroxiredoxin-2 (Prdx2) is a member of the highly homologous peroxiredoxin (Prdx) protein family [5]. Prdx2 expression is elevated in several human cancer cells and tissues and influences diverse cellular processes including survival, proliferation, and apoptosis [6,7,8]. The overexpression of Prdx2 rendered leukemia and stomach cancer cells resistant to various chemotherapeutic agents [9,10], and the downregulation of Prdx2 sensitized head and neck cancer cells to radiation and gastric carcinoma cells to cisplatin [11,12]. Furthermore, silencing of Prdx2 enhances the response of colorectal cancer cells to ionizing radiation and oxaliplatin [13]. Bioinformatics analysis has revealed that low Prdx2 expression was strongly correlated with poor survival in stomach cancer [14] and silencing of Prdx2 enhanced lung metastasis in melanoma [15]. Hence, Prdx2 is a key regulator of prognosis and treatment resistance in a variety of cancers.

Therefore, we investigated the relationship between Prdx2 and pNEN. In addition, we elucidated the role of Prdx2 in well-differentiated pNENs, and its utility as a predictive marker for everolimus response.

## 2. Materials and Methods 

### 2.1. Reagents and Materials

Everolimus (Sigma-Aldrich Inc., St. Louis, MO, USA) was diluted in dimethyl sulfoxide and stored at −20 °C. The following primary antibodies were used: Prdx1 (LF-MA0214; Ab Frontier, Seoul, Korea), Prdx2 (LF-MA0144; Ab Frontier, Seoul, Korea), Prdx-SO3 (LF-PA0004; Ab Frontier, Seoul, Korea), P-mTOR (#5536; Cell Signaling Technology, Danvers, MA, USA), mTOR (#2983; Cell Signaling Technology), p-ERK (#4377; Cell Signaling Technology), ERK (#9102; Cell Signaling Technology), p-AKT (#4060; Cell Signaling Technology), AKT (#4691; Cell Signaling Technology), Oct4 (ab18976; Abcam, Cambridge, UK), Sox2 (#4900S; Cell Signaling Technology), CD24 (sc-11406; Santa Cruz Biotechnology Inc., Santa Cruz, CA, USA), and glyceraldehyde 3-phosphate dehydrogenase (GAPDH) (sc-47724; Santa Cruz Biotechnology Inc., Dalas, TX, USA). Horseradish peroxidase-conjugated anti-rabbit and anti-mouse immunoglobin G was obtained from Millipore (AP132P, AP124P; Billerica, MA, USA).

### 2.2. Cell Lines and Cell Culture Conditions

Human pancreatic neuroendocrine cells were obtained from the Japanese Collection of Research Bioresources Cell Bank. The cells were cultured in Roswell Park Memorial Institute medium supplemented with 10% fetal bovine serum (Gibco, Thermo Fisher Scientific., Waltham, MA, USA) and 1% penicillin–streptomycin, and maintained at 37 °C in a humidified incubator under 5% CO_2_.

### 2.3. Spheroid Culture

Cells were maintained in Dulbecco’s modified Eagle’s medium/F-12 containing B-27 supplement (Gibco and Invitrogen, Carlsbad, CA, USA), N-2 supplement (17504-044 Gibco, Thermo Fisher Scientific., MA, USA), 1% penicillin–streptomycin, 50 ng/mL basic fibroblast growth factor (R&D Systems, Minneapolis, MN, USA), and 50 ng/mL epidermal growth factor (R&D Systems, Minneapolis, MN, USA) and cultured on an ultra-low-attachment plate at 37 °C under 5% CO_2_.

### 2.4. Cell Viability Assay

Cells (1 × 104/well) were seeded in a 96-well plate. After incubation for 24 h, increasing concentrations of fluorouracil were added. Subsequently, 20 μL of water-soluble tetrazolium salt solution (Ez-Cytox; DoGenBio, Seoul, Korea) was added to each well, and the cells were incubated at 37 °C under 5% CO_2_, for 3 h. Finally, the absorbance at 450 nm was measured using a microplate reader (E-MAX; Molecular Devices, San Jose, CA, USA).

### 2.5. Quantitative Reverse Transcription and Polymerase Chain Reaction (qRT-PCR)

In brief, purified total RNA was converted into first-strand cDNA using RNAiso Plus (TaKaRa Bio Inc., Shiga, Japan) and the RevertAid First Strand cDNA Synthesis Kit (Thermo Fisher Scientific, Waltham, MA, USA). First-strand cDNA of the target gene was amplified using specific primers with PowerUp™ SYBR™ Green Master Mix (Thermo Fisher Scientific., Waltham, MA, USA) on an Applied Biosystems StepOnePlus™ platform (Thermo Fisher Scientific., Waltham, MA, USA).

The following primer pairs were used: human GAPDH (NM-001357943.2), 5′-AGG GCT GCT TTT AAC TCT GGT-3′ and 5′-CCC CAC TTG ATT TTG GAG GGA-3′; human Prdx1 (NM-001202431.2), 5′-GGT TGC AGT AAG CCA ACA CC -3′ and 5′-CCT GAA GAC ATC TTC CTA TCA GC-3′; human Prdx2 (NM_005809.6), 5′-CAG ACG AGC ATG GGG AAG -3′ and 5′-ACG TTG GGC TTA ATC GTG TC-3′; human mTOR (NM_001386500.1), 5′-GCA GCT GCA TGG GGT TTA-3′ and 5′-CCC GAG GGA TCA TAC AGG T-3′; human ERK (NM_002745.5), 5′-CAA AGA ACT AAT TTT TGA AGA GAC TGC-3′ and 5′-TCC TCT GAG CCC TTG TCC T-3′; human AKT (NM_001014431.2), 5′-GGC TAT TGT GAA GGA GGG TTG-3′ and 5′-TCC TTG TAG CCA ATG AAG GTG-3′; human Sox2 (NM_003106.4), 5′-TGC GAG CGC TGC ACA T-3′ and 5′-GCA GCG TGT ACT TAT CCT TCT TCA-3′; human Oct4 (NM_001173531.3), 5′-GGG CTC TCC CAT GCA AAA C-3′ and 5′-CAC CTT CCC TCC AAC CAG TTG C-3′. Target gene expression was normalized to that of GAPDH. Relative gene expression was set to one-fold for the untreated control, and the normalized fold-change ratio was calculated using the 2−ΔΔCt method.

### 2.6. Transfection of siRNA

We purchased siRNAs against NFE2L2 from Dharmacon (Thermo, Lafayette, CO, USA). The siRNAs were dissolved in RNase-free H2O, diluted, and transfected into cells using Lipofectamine RNA iMAX™ (Invitrogen, Calsbad, CA, USA). A scrambled siRNA was used as the negative control.

### 2.7. Measurement of ROS

Intracellular ROS levels were measured using H2DCF-DA (2′,7′-dichlorodihydrofluorescein diacetate) dye (Invitrogen, Calsbad, CA, USA). At the indicated times, dye was added to the culture medium to a final concentration of 50 μM. After incubation for 15 min, flow cytometry was performed using a BD FACS CANTOII flow cytometer (BD Biosciences, Durham, NC, USA).

### 2.8. Immunohistochemistry

Paraffin sections were stained with hematoxylin and eosin. For the immunohistochemical assay, deparaffinized sections were subjected to antigen retrieval by heating in sodium citrate buffer (pH 7.0). The anti-Prdx2 primary antibody (LF-MA0144; Ab Frontier, Seoul, Korea) diluted to 1:100 was added, and the sections were incubated overnight at 4 °C. Finally, the sections were photographed under a light microscope at 400× magnification.

### 2.9. Sphere Formation Assay

A sphere formation assay was performed, as described previously. QGP-1 cells were isolated from culture medium, and single-cell suspensions (5000 cells/well) were plated in an ultra-low-attachment six-well plate and incubated for 14 days. The spheres were evaluated and counted under an inverted microscope.

### 2.10. In Vivo Study

Animal experiments were performed at the Center of Animal Care and Use at Gil Medical Center, Gachon University College of Medicine (Seongnam, Korea) according to institutional guidelines and with prior approval from the Institutional Animal Care and Use Committee (LCDI-2019-0156) and the Gil medical center ethics committee (GAIRB2014-268). We used the xenograft model for in vivo experiments, in which tumorigenic and spheroid QGP-1 cells develop in female BALB/c nude Foxn1 mice. Thirty female BALB/c nude Foxn1 mice (age, 5 weeks) were supplied by Orient Bio, Inc. (Seongnam, Korea). The mice were adapted to local conditions for 1 week, and then injected subcutaneously in the right flank with 1 × 10^7^ QGP-1 cells or 2.5 × 10^6^ QGP-1 spheroid cells in 100 μL of Matrigel. Five weeks after implantation, the mice were euthanized with CO_2_, and the tumors were excised and stored in liquid nitrogen for hematoxylin and eosin and immunohistochemical staining.

### 2.11. Statistical Analyses

Results are expressed as the means ± standard deviations of at least three independent experiments. Statistical comparisons were performed using one-way analysis of variance followed by Tukey’s post hoc test. Values of *p* < 0.05 were considered to indicate statistically significant differences. Graphs were prepared using Prism software version 8.0 (GraphPad Software Inc., La Jolla, CA, USA).

## 3. Results

### 3.1. Prdx2 Expression is Upregulated in QGP-1 pNEN Cells 

**Figure 1:**
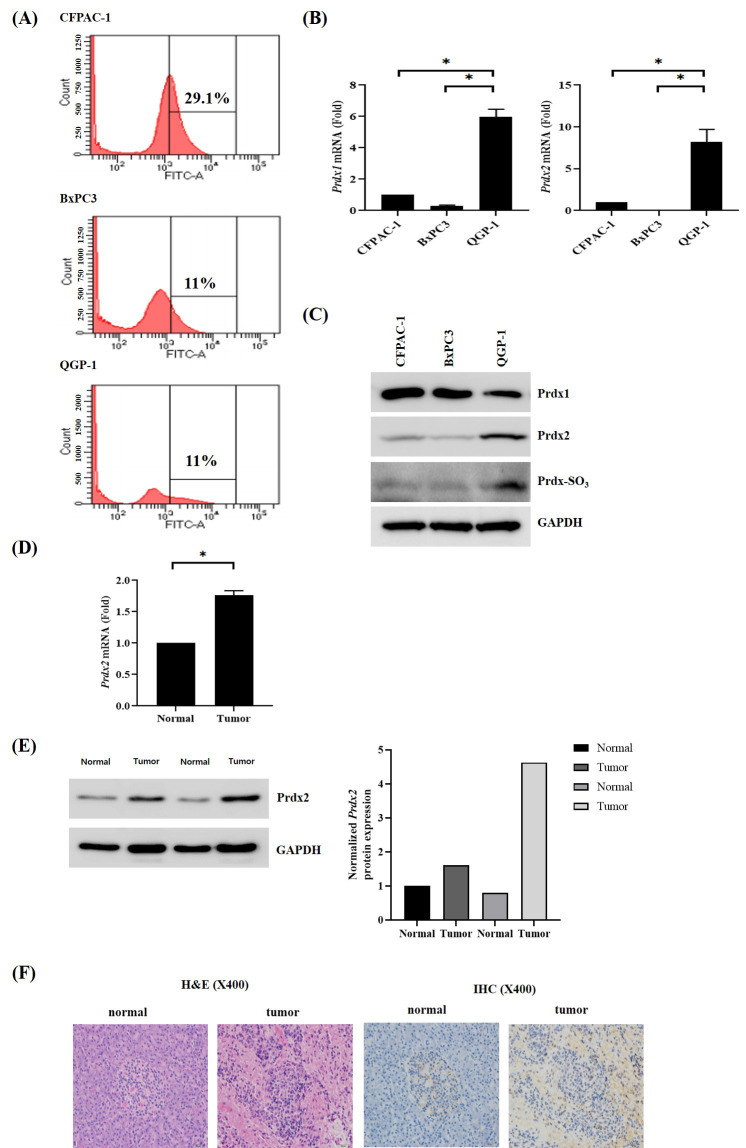
Peroxiredoxin-2 (Prdx2) overexpression in human pancreatic neuroendocrine neoplasms. (**A**) Intracellular reactive oxygen species (ROS) levels were lower in pancreatic neuroendocrine neoplasm (QGP-1) cells than in pancreatic cancer cells (BxPC3, CFPAC) based on 2’,7’-dichlorofluorescein diacetate (H2DCFDA) staining; (**B**) The mRNA levels of Prdx1 and Prdx2 were higher in QGP-1 cells than in pancreatic cancer cells (BxPC3, CFPAC-1); (**C**) Western blotting showed that Prdx2 levels increased in QGP-1 cells as compared with pancreatic cancer cells (BxPC3, CFPAC); (**D**) Results of quantitative reverse transcription and polymerase chain reaction analysis comparing Prdx2 expression between pancreatic neuroendocrine tumors and adjacent normal pancreas in two patients; (**E**) Western blotting showed that Prdx2 was upregulated in pancreatic neuroendocrine tumors as compared with adjacent normal pancreas in two patients; (**F**) Representative immunohistochemical staining of Prdx2 in human pancreatic neuroendocrine neoplasm tissue and normal pancreatic tissue showing the strong staining of Prdx2 in tumor tissue. Magnification × 400. * *p* < 0.05.

Prdx2 expression was elevated and intracellular reactive oxygen species (ROS) levels were reduced in QGP-1 cells as compared with control pancreatic ductal adenocarcinoma (PDAC) cells (BXPC3 and CFPAC) (Figure 1A,B). Western blotting revealed that Prdx1 was downregulated and Prdx2 was overexpressed in QGP-1 cells with higher levels of Prdx-SO3 (Figure 1C). Immunohistochemical (IHC) staining of human pNEN tissue from patients who underwent surgical resection revealed elevated expression of Prdx2 in pNENs as compared with normal pancreatic tissue from the same patients (Figure 1D–F). 

### 3.2. Everolimus Downregulates mTOR but Upregulates MAPK/ERK Pathway in QGP-1

**Figure 2:**
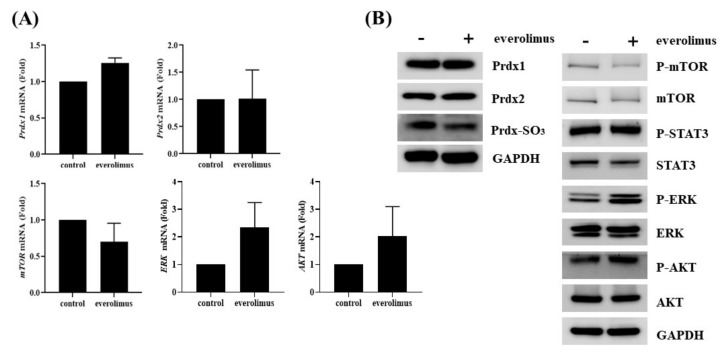
Effects everolimus on QGP-1 cells. (**A**) Everolimus downregulated mTOR expression and upregulated that of ERK and AKT in QGP-1 cells; (**B**) Western blotting showed that the levels of mTOR and phosphorylated mTOR decreased, whereas those of Prdx1, Prdx2, and Prdx-SO3 were unchanged in QGP-1 cells.

After everolimus treatment in QGP-1 cells, downregulation of mTOR expression and upregulation of ERK and AKT expression were observed. (Figure 2A). Western blotting revealed an increase in phosphorylated form of ERK and AKT after everolimus treatment, without altering Prdx expression or Prdx-SO3 levels (Figure 2B). 

### 3.3. Silencing of Prdx2 Increased Resistance to mTOR Inhibitors by Activating Proliferation-Related Signaling Pathways

**Figure 3:**
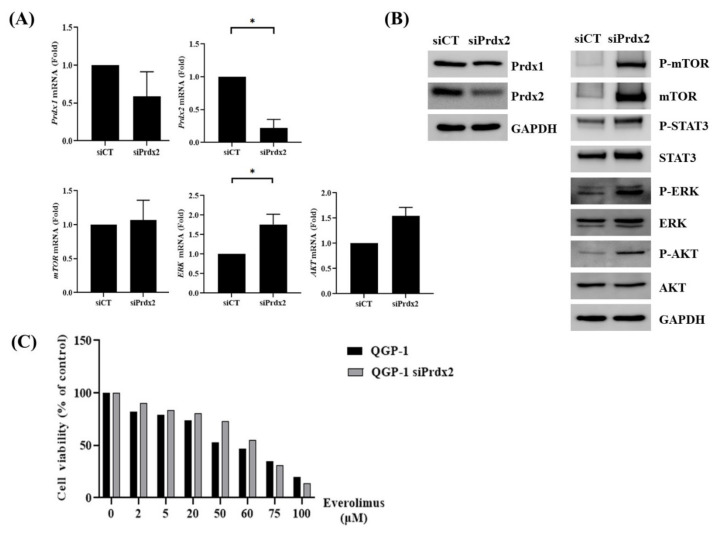
Effect of Prdx2 knockdown on QGP-1 cells. (**A**) The mRNA level of Prdx2 was downregulated, and that of ERK was upregulated, in siPrdx2 QGP-1 cells; (**B**) Western blotting showed that downregulation of Prdx2 was achieved partially, but the level of mTOR, phosphorylated mTOR, phosphorylated ERK, and phosphorylated AKT were increased in siPrdx2 QGP-1; (**C**) MTT assay showed that the antitumor effect of everolimus was significantly lower in siPrdx2 QGP-1 cells than in the negative control cells. * *p* < 0.05.

Knockdown of Prdx2 by small interfering RNA (siRNA) induced overexpression and phosphorylation of ERK and AKT (Figure 3A,B). In addition, siPrdx2 induced resistance to everolimus, as indicated by an increased IC50 value (Figure 3C).

### 3.4. Expression of Prdx2 is Downregulated in QGP-1 Spheroid Cells

**Figure 4:**
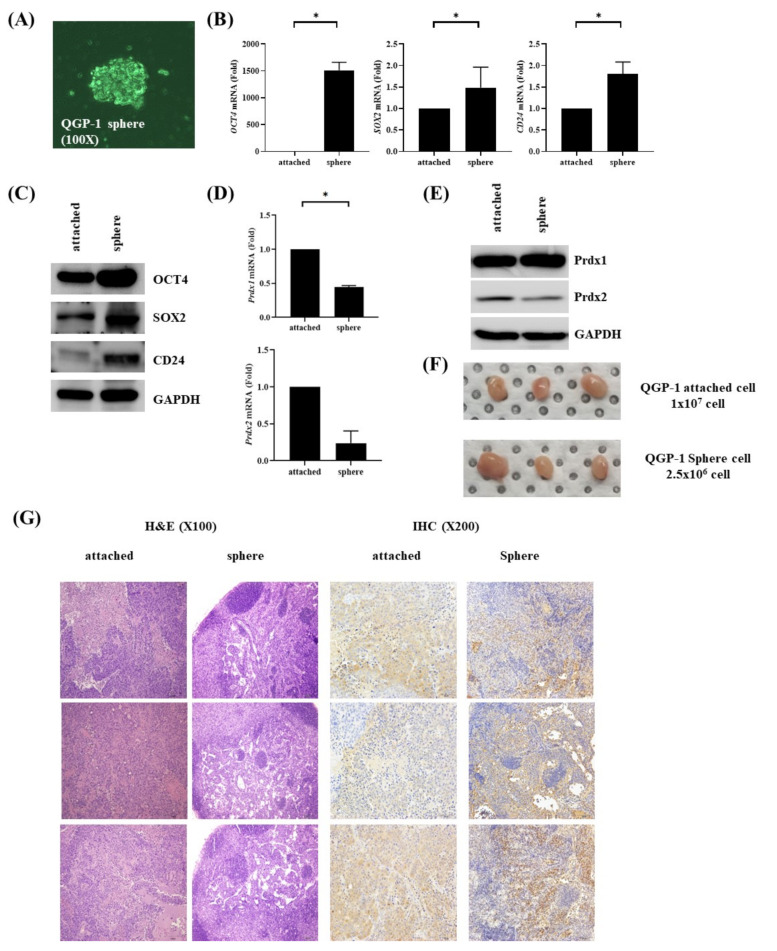
Prdx2 downregulation in pancreatic neuroendocrine tumor QGP-1 spheres. (**A**) QGP-1 spheres cultured via sphere formation assays; (**B**) The mRNA levels of Oct4, SOX2, and CD24 were upregulated in QGP-1 spheres as compared with the controls; (**C**) Western blotting revealed that OCT4, SOX2, and CD24 levels were higher in QGP-1 spheres than in the controls; (**D**,**E**) Prdx2 was downregulated in QGP-1 spheres as compared with the controls at the mRNA and protein levels; (**F**) In vivo tumor cell xenograft assay showed that injection of QGP-1 spheres (2.5 × 10^6^ cells) resulted in the formation of a tumor comparable to that generated by QGP-1 cells (1 × 10^7^); (**G**) Immunohistochemical staining of Prdx2 in xenografted tumor showing weak staining for Prdx2 in QGP-1 sphere cells with small to medium sized nests. Magnification H&E ×100, IHC × 200. * *p* < 0.05.

QGP-1 spheroid cells exhibited cancer stem cell-like features, including expression of stem cell markers such as OCT4, SOX2, and CD24 (Figure 4A–C). Interestingly, Prdx2 was downregulated in QGP-1 spheroid cells at the RNA and protein levels (Figure 4D,E).

### 3.5. Xenograft Model for QGP-1 and QGP-1 Spheroid Cells

QGP-1 and QGP-1 spheroid cells were planted in nude mice, resulting in the establishment of xenograft tumors. QGP-1 spheres, which exhibited cancer stem cell-like features in vitro, generated comparably sized xenograft tumors despite the smaller number of cells (2.5 × 10^6^ vs. 1 × 10^7^, Figure 4F). IHC staining of xenograft tumors generated by QGP-1 sphere revealed the downregulation of Prdx2 forming small to medium sized nests (Figure 4G).

## 4. Discussion

The expression of Prdx2 was upregulated in QGP-1 and patient-derived pNEN cells. Whereas Prdx1 was downregulated, Prdx-SO3 was still increased in protein level of QGP-1 representing that Prdx2 can be more sensitive to hyperoxidation than Prdx1, which is concordant with previously published reports [16,17] Knockdown of Prdx2 in QGP-1 cells activated proliferation-related signaling pathways and increased resistance to mTOR inhibitors. These findings suggest a role for Prdx2 as a suppressor of pNENs and for predicting the therapeutic response of pNENs to mTOR inhibitors such as everolimus. Interestingly, QGP-1 spheroid cells, which exhibited cancer stem cell-like features in vitro and in a xenograft model, expressed low levels of Prdx2 and exhibited low activation of mTOR-related signaling pathways.

PDAC is aggressive, resistant to chemotherapy, and is associated with poor survival with rapid progression and metastasis. By contrast, pNENs have clinical characteristics different from those of PDACs, i.e., indolent progression and metastasis. Because nonfunctioning pNENs are usually asymptomatic, many patients are diagnosed at a metastatic stage, and the pNEN may exhibit aggressive features even if the primary tumor is small (<2 cm) [18]. Whereas the microenvironment of PDAC is characterized by hypovascularity and abundant extracellular matrix, which hamper the delivery of chemotherapeutic agents to cancer cells, pNENs are hypervascular, and systemic antitumor agents delivered via the bloodstream can be effective for metastatic or aggressive pNENs.

In general, mTOR inhibitors, tyrosine kinase inhibitors, and systemic chemotherapy are used to treat pNEN. However, the therapeutic effectiveness of the available systemic agents is suboptimal; they result in progression-free survival of approximately 11 months [4,19]. Nevertheless, the low incidence of pNEN makes it impossible to conduct large clinical trials to validate the effectiveness of antitumor agents. Furthermore, there are few sources of cell lines and in vitro models of pancreatic neuroendocrine tumors, which hampers the discovery of novel therapeutic agents and therapeutic markers [20].

Prdx is an ROS scavenger and plays a role in proliferation and epithelial-mesenchymal transition (EMT) in various cancers, including breast cancer [21], colorectal cancer [22,23], prostate cancer [24], and melanoma [15]. Downregulation of Prdx2 has been reported to correlate with increased proliferative and migratory activities in melanoma cell lines and in vivo melanoma models [15]. In addition, Prdx2 inhibited EMT in a colorectal cancer model, reducing invasive characteristics, via Twist1, Snail, ZEB1, and ZEB2 [25]. These findings suggest a suppressive role for Prdx2 in cancer proliferation and EMT. However, Prdx2 has been shown to increase nodal metastasis in lung cancer cells [26], and a positive correlation between Prdx2 and chemoresistance in breast and pancreatic cancer has been reported [27,28]. Additionally, it has been reported that there is a direct physical interaction between collapsing response mediator protein 2 (CRMP2) which regulates microtubule structure during migration or neuronal development and cytoplasmic Prdx2 in Jurkat T-lymphoma cells [29]. Therefore, Prdx2 may play different roles according to cancer type and our data provide insight into the role of Prdx2 in pNEN.

In this study, everolimus inhibited the mTOR and upregulated mitogen-activated protein kinase (MAPK/ERK) signaling pathways in QGP-1 cells. Interestingly, even partial downregulation of Prdx2 in QGP-1 cells activated the MAPK/ERK signaling pathway and increased resistance to everolimus. This is concordant with previous reports of crosstalk between the mTOR signaling pathway and other proliferation-related pathways including the MAPK/ERK pathway [30,31,32].

The mechanisms of crosstalk between signaling pathways can be grouped into four categories including negative feedback loop, cross-inhibition, cross-activation, and pathway convergence [30]. Cross-inhibition can be validated when one signaling pathway is inactivated and the other pathway is effectively activated. Ras/ERK and PI3K/AKT pathways are good examples of this type of negative regulation. In one study, which validated the crosstalk between MAPK and PI3K/mTOR pathways using twenty-nine cancer cell lines from different origin, synergistic interaction between pathways were shown in cell lines with PTEN loss [33]. Another study also reported that the inhibition of mTORC1 leads to MAPK pathway activation through PI3K-dependent feedback loop in vitro and in vivo model. Interestingly, RAD001 treatment in PTEN-null prostate conditional mice led to MAPK activation and PTEN could be the key molecule that could drive the crosstalk between pathways [32].

ROS are known to affect the growth factor signaling pathway; PTEN, which is known as a tumor suppressor, is affected by oxidative stress [34]. H_2_O_2_ produced by growth factor-stimulated cells activates PIP3 and AKT, and also inactivates PTEN through oxidation. In this process, Prdx2, a well-known ROS scavenger, seems to have a pivotal role in PTEN oxidation. The increased local concentration of H_2_O_2_, on the one hand, by inactivation of Prdx2 inhibits the activity of PTEN by oxidizing the catalytic cysteine residue and increased PIP3 triggering downstream signaling pathways. Reactivation of Prdx subsequently decreased H_2_O_2_ concentration and oxidized PTEN was reactivated [34]. On the basis of these data, co-inhibition of multiple signaling pathways is under investigation for multiple cancer types [35,36]. Some clinical trials have attempted to block the mTOR and MAPK/ERK pathways to overcome adaptive resistance [37,38]. 

Our result suggests that the crosstalk between mTOR pathway and MAPK/ERK pathway seems to work in pNEN as well. Especially in the situation of low Prdx2 expression, inhibition of the mTOR pathway activated the MAPK/ERK pathway resulting in induction of mTOR inhibitor resistance. The expression level of Prdx2 may enable estimation of mTOR inhibitor responsiveness as a potential surrogate therapeutic efficacy marker or can be used as a target to overcome resistance to mTOR inhibitors. However, further studies are needed to confirm that Prdx2 overexpression enhances the efficacy of mTOR inhibitors or re-sensitizes mTOR inhibitor-resistant pNEN.

Although the role of cancer stem cells in tumorigenesis is acknowledged in many types of cancers, pNEN cancer stem cells have recently emerged as a therapeutic target [39]. Interestingly, in QGP-1 spheroid cells in which stem cell markers were overexpressed, Prdx2 was downregulated, as was the mTOR signaling pathway, one of the main targets of current therapeutic agents for pNEN. These findings suggest that pNEN stem cell proliferation may not benefit from mTOR inhibitors alone and may justify combination therapy with multiple agents that inhibit the proliferation of both cancer cells and cancer stem cells in which mTOR signaling is inactive. Ongoing phase I–II trials are investigating the efficacy of combination therapy with systemic mTOR inhibitors such as everolimus and erlotinib, a tyrosine kinase receptor inhibitor targeting EGFR mutation (NCT00843531) or cixutumumab, a human monoclonal antibody targeting type 1 human insulin-like growth factor receptor, everolimus, and octreotide (NCT01204476). Although the outcomes of those trials are not yet available, the clinical benefits of these combination therapies will be announced soon, and our data may provide a mechanism for their effects.

This study had the following limitations. First, because in vitro models and cell lines are scarce, only QGP-1 cells were used. However, the overexpression of Prdx2 was validated in pathologically proven human pNEN tissue. Second, the efficacy of Prdx2 overexpression in QGP-1 spheroid cells was not validated and the relationship between Prdx2 expression and cancer stem cell proliferation was not confirmed. Further studies are needed to confirm the relationships among the expression level of Prdx2, cell-signaling pathways, and the proliferation of pNEN stem cells.

## 5. Conclusions

Prdx2 may suppress the growth of pancreatic neuroendocrine neoplasms and could be a marker for predicting the efficacy of mTOR inhibitors. Further studies of the therapeutic potential of Prdx2 in pNEN are warranted.
